# Detergent-Resistant Membranes in Chloroplasts and Mitochondria of the Halophyte *Salicornia perennans* under Salt Stress

**DOI:** 10.3390/plants12061265

**Published:** 2023-03-10

**Authors:** Olga A. Rozentsvet, Elena S. Bogdanova, Vadim N. Nurminsky, Viktor N. Nesterov, Michael Yu. Chernyshov

**Affiliations:** 1 Samara Federal Research Scientific Center RAS, Institute of Ecology of Volga River Basin RAS, Russian Academy of Sciences, 10, Komzin St., 445003 Togliatti, Russia; 2 Siberian Institute of Plant Physiology and Biochemistry, Siberian Branch, Russian Academy of Sciences, 132, Lermontov St., 664033 Irkutsk, Russia; 3 Presidium of Irkutsk Scientific Center, Siberian Branch, Russian Academy of Sciences, 134, Lermontov St., 664033 Irkutsk, Russia

**Keywords:** chloroplasts, detergent-resistant membrane, lipids, mitochondria, protection against salt stress

## Abstract

Halophytes represent important models for studying the key mechanisms of salt tolerance. One approach to the development of new knowledge of salt tolerance is to study the properties of detergent-resistant membranes (DRMs). In this work, the lipid profiles of DRMs of chloroplasts and mitochondria of euhalophyte *Salicornia perennans* Willd, before and after their exposure to shock concentrations of NaCl, have been investigated. We found that DRMs of chloroplasts are enriched in cerebrosides (CERs) and that sterols (STs) dominate the mass of mitochondrial DRMs. Also, it has been proven that (i) the impact of salinity provokes obvious growth in the content of CERs in DRMs of chloroplasts; (ii) the content of STs in DRMs of chloroplasts does not change under the influence of NaCl; (iii) salinity also causes some elevation in the content of monounsaturated and saturated fatty acids (FAs). Considering the fact that DRMs represent integral parts of both chloroplast and mitochondrial membranes, the authors have come to the conclusion that the cells of euhalophyte *S. perennans*, under the impact of salinity, presumes the choice (by the cell) of some specific composition of lipids and FAs in the membrane. This may be considered as a specific protection reaction of the plant cell against salinity.

## 1. Introduction

The problem of salinity is vitally important because many soils in the world are saline. It is necessary to solve the problem of the survival of plants under extreme conditions and at extreme locations [1]. No wonder the practical need to protect agricultural plants from salinity has attracted the attention of many researchers, who have initiated investigations in order to understand the mechanisms of plants’ protection at the level of the plant cell.

In order to approach solving the problem of salt tolerance, it is necessary to understand the processes of regulation of functions of cell organelles and ion fluxes in plants growing on saline soils. The problem of salt stress tolerance was discussed, for example, for halophytes in the aspects of (i) violation of homeostasis of *reactive oxygen species* (ROS) in halophytes [2], (ii) chloroplast function and ion regulation in plants growing on saline soils [3], (iii) and vesicular transport of Na^+^ and Cl^−^ as the mechanism of salt tolerance in halophytes [4].

Any approaches to salt tolerance are of interest. These may include the issue of multiple compartmentalization of sodium, which can grant salt tolerance for some time [5], or the issue of salt stress sensing and early signaling [6]. It was discovered that sphingolipids react to salt by triggering Ca^2+^ influx into the system [7]. Furthermore, the issue of the functions of biological membranes and membrane microdomains, which can take part in the processes of protection from salinity, and also the issue of related potential capacities of the membranes were discussed. The authors of the present paper studied the “Adaptive Mechanisms of Halophytes and Their Potential in Improving Salinity Tolerance in Plants” [8], and this stimulated them to undertake deeper investigations of cellular processes and processes in organelles and investigations of the structures, functions, and biochemistry of plant membranes.

J. D. Nickels and co-authors investigated the in vivo structure of biological membranes known to be characterized by domain organization; this team of authors interpreted the cell membrane as some mosaic of discrete microdomains [9]. In some publications, discrete microdomains were interpreted as either detergent-resistant fractions of the plasma membrane [10] or as detergent-resistant membrane microdomains [11].

Meanwhile, with time most of the specialists in plant biology have already discussed *detergent-resistant membrane microdomains* (DRMs), which have been differently interpreted in publications, for example, as lipid rafts located in endomembranes [12], e.g., in the endomembranes of wild halophytes [13]. Note that D. A. Brown was sure that DRMs did not represent rafts [14]. Furthermore, DRMs were revealed to exist not only in plasma membranes but also in vacuolar membranes [15,16].

In the investigations researching lipid microdomains, the researchers traditionally used the technique of isolation of detergent-resistant membranes [17,18,19,20]. For example, the issue of isolation of nano–meso-scale detergent-resistant membranes was discussed by R. J. Morris and colleagues [19].

The popularity of the conception of membrane microdomains led to the appearance of new points of view on DRMs. According to the known assumptions, membrane microdomains could be found in the inner parts and the outer parts of plasma membranes or vacuolar membranes. DRMs were supposed to form so-called functional platforms, considered to be likely needed in the regulation of some special cellular functions [12,21]. DRMs interpreted as rafts were discussed as being involved in such cellular processes as signaling, trafficking [22,23] and regulatory processes [23,24,25], in the transport of nutrients [26] and in vesicular transport [4], etc.

Lipids of DRMs contain not only sterols (STs) and sphingolipids (SLs) or cerebrosides (CERs), but also glycerolipids with saturated fatty acids (FAs) [27]. A high degree of saturation of the lipids with FAs, acceptor, and donor groups of CERs, and the tetracyclic sterane ring of STs aid in the dense packing of these lipids and in their resistance with respect to their solubilization by non-ionic detergents [17,18].

In earlier investigations by our team, we revealed some biochemical markers of DRMs in chloroplast membranes and in mitochondrial membranes of leaves of euhalophyte *Salicornia perennans* and glycohalophyte *Artemisia santonica* [13,28]. The effect of salt stress upon the lipid profile of DRMs of halophyte cell membranes was the objective of our further investigation. Furthermore, some of our earlier investigations allowed us to conclude that the protection of the plant cell against salt stress may be bound up with the capability of the cell to vary the composition of lipids in definite microdomains.

Thus, the objective of our investigation relates to the study of the chloroplasts and mitochondria of euhalophyte *S. perennans* under the conditions of salt stress. The present paper will discuss variations in the lipid profile in DRMs of chloroplasts and mitochondria of *S. perennans* under salt stress to the end of understanding the mechanism of protection of the plant cell against salt stress.

## 2. Results

The lipid profile of detergent-resistant membranes of chloroplasts and mitochondria of euhalophyte *S. perennans* before and after their exposure to shock concentrations of NaCl has been investigated by our team.

**Figure 1:**
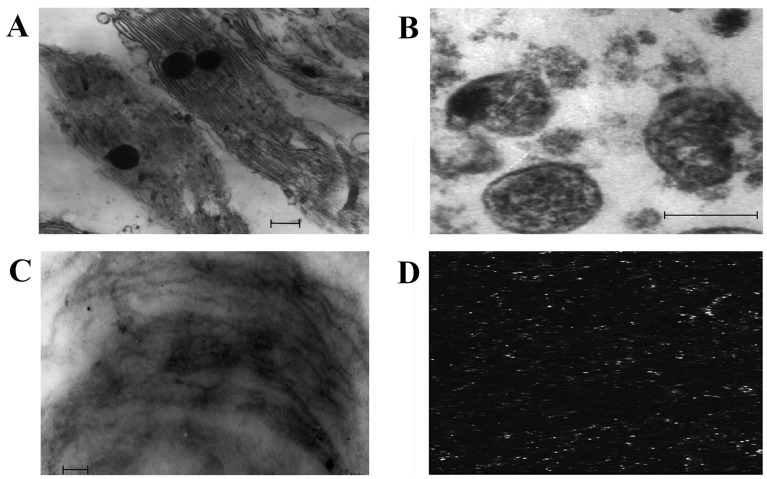
Electron microscopy of chloroplasts (**A**), mitochondria (**B**), DRMs of chloroplasts (**C**), confocal microscopy of DRMs of mitochondria (**D**). Scale bars = 0.2 µm.

To obtain more information on membrane lipids, we isolated DRMs from chloroplasts and mitochondria. Processing with detergent (Triton X-100) and subsequent centrifugation of the membrane material in the sucrose density gradient allowed us to identify the bright opalescent zone within the band of 15% sucrose, which is one of the criteria for DRM presence. The data from electron and confocal microscopy obtained during our experiments demonstrated the state of the chloroplast membranes before and after exposure to the detergent (Figure 1). For example, before the influence of the detergent, strictly structured chloroplasts were revealed as having a system of thylakoid membranes (Figure 1A). After the effect of the detergent and the identification of the opalescent zone of chloroplast membrane material in the sucrose density gradient, only thread-shape structures were revealed. These represented the membranes remaining stable following the addition of the detergent (Figure 1C). Figure 1B demonstrates the mitochondrial fraction isolated from the native cells of *S. perennans*. The application of the fluorescent probe laurdan allowed us to reveal DRMs in mitochondria (Figure 1D).

The detailed analysis of chloroplast and mitochondrial membrane lipid profiles revealed substantial differences in comparison to the lipid content of the respective DRMs.

**Figure 2:**
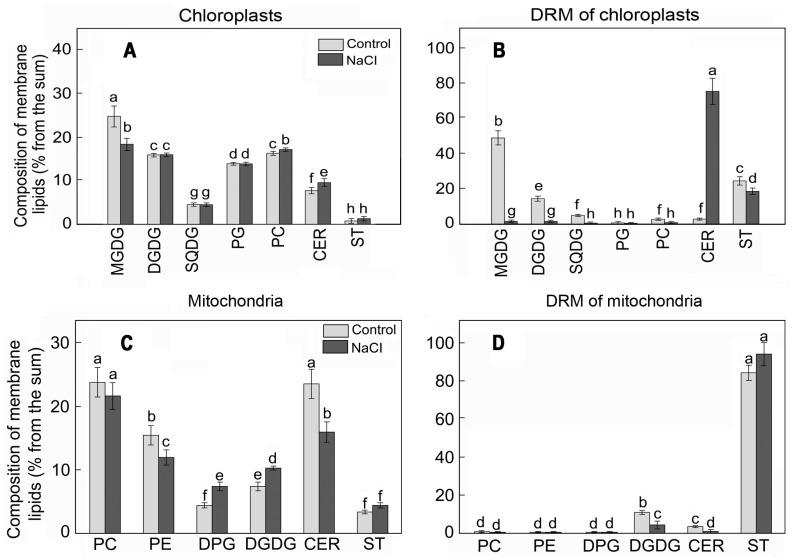
The lipid profiles of chloroplast (**A**) and mitochondrial (**C**) membranes and respective DRMs (**B**,**D**) of *S. perennans* before and after exposure to salinity (NaCl) stress. MGDG, monogalactosyldiacylglycerol; DGDG, digalactosyldiacylglycerol; SQDG, sulfoquinovosyldiacylglycerol; PG, phosphatidylglycerol; PC, phosphatidylcholine; phosphatidylethanolamine (PE); DPG, diphosphatidylglycerol; CERs, cerebrosides; STs—sterols. The data were obtained by the Mann–Whitney U test technique (*p* < 0.05) and represent M ± SD of three biological replicates. Letters above the bars indicate statistical meaning (*p* < 0.05).

For example, the main lipids of *S. perennans* chloroplasts were represented by monogalactosyldiacylglycerol (MGDG) and digalactosyldiacylglycerol (DGDG), while the share of CERs and STs did not exceed 10% of the total mass of lipids (Figure 2A). In DRM lipids of chloroplasts, the total mass of STs and CERs was 30%; furthermore, the content of STs was higher in comparison to the content of CERs (Figure 2B). In contrast to chloroplast membranes, phosphatidylcholine (PC) and phosphatidylethanolamine (PE) were the principal lipids in mitochondrial membranes (Figure 2C). The share of CERs and STs in mitochondrial membranes was about 20%, and the share reached 80% for the DRMs of mitochondria (Figure 2D).

The effect of NaCl led to reorganization in the composition and the content of chloroplast lipids: lowering the level of glycolipids (mainly MGDG), growth of the concentration of phospholipids, and small elevation of the share of CERs. More substantial variations of the content were observed in DRMs of chloroplasts, i.e., multiple growths in the proportion of CERs at the background of lowering of concentrations of all other lipid components (Figure 2B). A different situation was observed in mitochondrial DRMs: STs were the principal structural components of DRMs in them and the content of STs practically did not change under the influence of salinity (Figure 2D).

**Figure 3:**
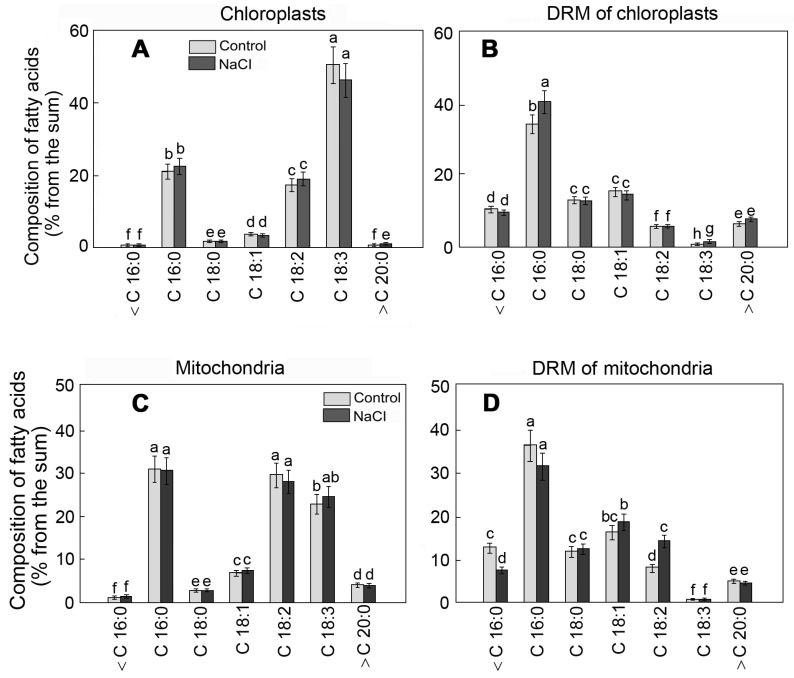
The FA composition of chloroplast (**A**) and mitochondrial (**C**) membranes and respective DRMs (**B**,**D**) of *S. perennans* before and after exposure to salinity (NaCl) stress. <C16:0—the sum of short-chain FAs; C16:0—palmitic acid; C18:0—stearic acid; C18:1—oleic acid; C18:2—linoleic acid; C18:3—linoleic acid; >C20:0—the summary value of long-chain FAs; The data were obtained by the Mann–Whitney U test technique (*p* < 0.05) and represent M ± SD of three biological replicates. Letters above the bars indicate statistical meaning (*p* < 0.05).

The specific fatty acid content of lipids is an essential element of rafts. According to our results, the portion of unsaturated fatty acids in chloroplast membranes was 75%, and linolenic acid (C18:3n3) was the dominant form (Figure 3A). The content of FAs in DRMs, before and after exposure to salinity, was characterized by the growth of the relative content of palmitic acid (C16:0) from 34 to 40% (Figure 3B).

Unlike that in chloroplasts, linoleic acid (C18:2n6) was the dominant unsaturated fatty acid in mitochondrial membranes (Figure 3C). The portion of saturated palmitic acid was 33%. The influence of NaCl led to the growth of the content of C 18:2 in DRM by 1.8 times (Figure 3D). Meanwhile, the contents of FAs, both in the organelle membranes and in respective DRMs, varied under the effect of salinity to a lesser extent compared to the membrane lipids.

## 3. Discussion

Halophytes were known to represent convenient models for studying the mechanisms of salt tolerance [1,4]. It was important for our team to study the capability of DRM of halophyte cells to respond to salt stress. The capability to respond to stress factors (possibly by varying the lipid content of DRMs) represented a very important property of plant cells. According to the literature data, DRMs of plant cells are able to respond, for example, to abiotic types of stress (temperature stress [10,16,29], osmotic stress [16,24,25], or the action of some pathogens [25]).

The data from electron and confocal microscopy allowed us to observe the evidence of DRM presence in such cell organelles of halophyte *S. perennans* as chloroplasts and mitochondria.

The main indicator that distinguished DRMs from the native mitochondrial and chloroplast membranes of halophyte *S. perennans* was the enrichment of the composition of STs and/or CERs. In combination with resistance to the effect of Triton-X 100, these data provided additional evidence of the existence of DRMs, not only in the plasma membrane but also in other cell compartments. The results of our investigations into mitochondria and chloroplasts appeared to be consistent with the results of similar investigations conducted by other researchers into *Arabidopsis thaliana* and *Allium porrum*. It was earlier shown that the concentration of CERs in the plasma membrane DRMs was 4–5 times higher than in the plasma membrane itself, in microsomal membranes and in Golgi membranes [18,30].

The functional activity of DRMs involved in reactions to environmental stress factors is likely to be supported by some definite contents of a definite set (composition) of the lipids and fatty acids in DRMs. For example, it is known that the envelope of chloroplasts is predominantly formed by phosphatidylcholine (PC), phosphatidylglycerol (PG), phosphatidylinositol (PI), as well as monogalactosyldiacylglycerol (MGDG) and digalactosyldiacylglycerol (DGDG) [23]. STs and CERs are present in the envelope of chloroplasts in small percentages (<2–3%) [28]. The thylakoids are formed mainly of glycolipids MGDG, DGDG, sulfur-containing lipid sulfoquinovosyldiacylglycerol (SQDG), and PG [23]. It is also known that mitochondrial membranes of plant cells (in addition to the two predominant classes of lipids, PC and PE contain phosphatidylglycerol (PG), phosphatidylinositol (PI), and mitochondria-specific diphosphatidylglycerol (DPG) [31]. STs and CERs are present in mitochondrial membranes in somewhat larger percentages (3–10%) than in membranes of chloroplasts [13].

The optimal growth and development of euhalophytes were known to take place under NaCl concentrations of no higher than 500 mM [5]. As it was ascertained in our investigation, the 1M concentration of NaCl formed stress conditions, under which some substantial growth of the content of CERs and saturated FA C16:0 was noted in DRM lipids of chloroplasts. In mitochondrial DRM lipids, the effect of NaCl had a minor influence on the content of the principal component, i.e., STs. Consequently, Na^+^ ions influenced the DRM composition of endomembranes, especially endomembranes of chloroplasts, and this was reflected to a somewhat larger extent in the content of CERs (in comparison to the content of STs). The modification of the lipid composition of the tonoplast in response to the elevation of salinity was previously reported [32]. The variations of the content of lipids in the DRMs of chloroplasts and mitochondria may represent a similar mechanism related to protection of the cell compartments (considered to be useful from the viewpoint of energy regulation and photosynthesis activity) against excessive salt penetration into the cytosol.

As shown in publications by the authors in our team, both STs and CERs were known to be capable of modulating some biophysical properties and some physiological functions of the membranes. This conclusion was in some sense confirmed by the results of D. Takahashi and coauthors in their work with low-temperature stress [10]. For example, variations in the ST:CER ratio in the plasma membrane during cold acclimatization led to changes in the thermodynamical properties and in the physiological functions of DRMs, which influenced plant resistance to low temperatures [10]. Meanwhile, under the conditions of our experiments bound up with salinity, the roles of STs and CERs in the protection of individual cell compartments of halophyte *S. perennans* differed substantially. So, it appeared obvious that the composition of DRM lipids could vary under the influence of stress factors.

It is known that membrane STs are considered one of the key links in the regulation of membrane ion permeability [27]. Experiments with the use of nystatin (specifically bound to STs) demonstrated (i) substantial growth of membrane permeability for water and K^+^, (ii) some elevation in the oxygen consumption by the cells, and (iii) some growth of the rate of heat release [29]. The stability of the elevated ST content in the DRMs of *S. perennans* mitochondria under salt stress was considered to be necessary for the regulation of ion transport fluxes and in the regulation of the level of ROS [33]. Furthermore, in contrast to that of mitochondria, the content of CERs is known to abruptly grow in DRMs of chloroplasts. Salt stress is known to elevate the ROS production in plants, especially in chloroplasts and peroxisomes [2,34].

Halophytes are known to use elevated levels of ROS in the capacity of salt stress signaling [8]. Investigations performed with *Arabidopsisthaliana* mutants, which were characterized by elevated sensitivity to salt and reduced Na^+^/H^+^ antiporter activity, allowed the researchers to understand the biochemical function of sphingolipids as sensors of monovalent cations and the role of sphingolipids in the regulation (and modulation) of signaling processes in the plasma membrane [7]. CERs, as one of the classes of sphingolipids, can provide similar functions in chloroplasts. It was also discovered that ROS can modulate ion fluxes through chloroplast membranes by activating the ion carriers [6].

It is worth adding that pathways of biosynthesis of STs and CERs intersect [22]. Usage of the enzyme 3-hydroxy-3-methylglutaryl-CoA reductase (HMGR), which triggers the biosynthesis of STs in *Arabidopsis thaliana*, results in the suppression of the biosynthesis of both STs and CERs [35]. Synthesis of FAs, as well as synthesis of STs and CERs, necessitates close interaction between the endomembranes. FAs are synthesized in chloroplasts, and, next, are either directly assembled in thylakoid lipids on the membranes or are exported into the ER for the purpose of assembly of extra-plastid lipids [23].

Grounding on the results of S. Shabala and coauthors [6], Z. Jiang and coauthors [7], E. Van Zelm and coauthors [8], the authors of the present paper have assumed that growth of the CER concentration in DRMs of euhalophyte *S. perennans* chloroplasts may also be bound up with the execution of both signaling and regulatory functions in response to elevated levels of NaCl.

## 4. Materials and Methods

### 4.1. Plant Materials

Euhalophyte *S*. *perennans* has become the object of our investigation. The seeds of the wild plants were collected at the end of October 2020 in the area of Prieltone (49°07′ N, 46°50′ E) and stored at room temperature for 6 months. The seeds were sprouted in distilled water in Petri dishes at 22–24 °C. The seedlings were transferred to containers with sand. Watering was carried out with Robinson nutrient solution (6 mM KNO_3_, 4 mM Ca(NO_3_)_2_, 2 mM MgSO_4_, 1 mM KH_2_PO_4_, 50 μM FeNa-EDTA, 50 μM H_3_BO_3_, 10 μM MnCl_2_, 1 μM ZnSO_4_, 0.5 μM CuSO_4_, 0.1 μM Na_2_MoO_4_) [36]. The plants were watered twice a week. Plant growth conditions were: air temperature of 20–22 °C, illuminance of 1200 µmol m^−2^ s^−1^, photoperiod of 10 h for 3 months. Afterwards, the plants were subdivided into the check group (further Control) and the experimental group (subjected to salinity, NaCl). The water solution of NaCl (1 M) was added once to the experimental plants. After 24 h the aboveground parts of the plants were cut off and used for isolation and analysis of membrane material. The check plants were subjected to similar manipulations.

The concentration (1 M NaCl) is stressful for halophytes and is chosen to demonstrate an effect at the level of DRM. Since, for example, euhalophytes in vivo tolerate 500 mM NaCl well, for the majority of cultivated plants, the concentration of 500 mm NaCl may be considered fatal.

### 4.2. Isolation of the Chloroplasts, Mitochondria and Their DRMs

The chloroplasts and mitochondria were isolated from the plant tissues according to the technique described in [13]. The fractions of organelles (~3 mg of protein) from halophyte formed the plant material, solubilized with the use of 1 mL of the buffer, which contained 1% of triton X-100, 10 mM Tris–HCl, 5 mM EDTA, 150 mM NaCl, 1 M of sucrose, and 1 mM PMSF, with a pH 7.5 for 30 min at 4 °C. Next, the yield of suspension was placed into the centrifuge tube and made up with 1 mL of 35% and 2 mL of each 25, 15, and 5% sucrose and centrifuged at 200,000× *g* (at 4 °C) for 2 h in the preparative ultracentrifuge (UP-65, MLW, Leipzig, Germany). The zone of opalescence of the DRMs was observed within the band of 15% of the sucrose density gradient [28].

### 4.3. Extraction and Analysis of the Lipids and Fatty Acids

The lipids were extracted from the subcellular fractions with the use of a chloroform/methanol mixture (1:2, *v/v*). Phospholipids, glycolipids, STs, and CERs were separated with the aid of high-performance thin-layer chromatography, as earlier described in [37]. The content of lipids was assessed densitometrically with the aid of available equipment (Denskan-04, Lenchrom, St. Petersburg, Russia). The chromatograms were analyzed in the parabolic approximation mode via the calibration curves. Methyl ethers of FAs were analyzed with the aid of gas–liquid chromatography in the isothermal regime (column temperature—180 °C; evaporator and detector temperature—260 °C; rate of carrying gas (helium)—2 mL min^−1^) in a Rtx T-2330 capillary column (length—105 m; internal diameter—0.25 mm; thickness of the immobile-phase film—0.25 mm) (RESTEK Corporation, Bellefonte, PA, USA); a chromatograph (Crystal-5000.1, Chromatech, Yoshkar-ola, Russia) was used. FAs were revealed by comparing their retention times with the standards (Supelco 37, Supelco, Bellefonte, PA, USA), and their quantification was performed with the use of the internal standard of heptadecanoate.

### 4.4. Electron and Confocal Microscopy

The chloroplasts and mitochondria were visually analyzed under the electron microscope JEM 100B (JEOL, Tokyo, Japan). The outer variations of the physical states of DRMs of the organelles were assessed with the aid of confocal microscopy (confocal luminescent scanning laser microscope MicroTime 200 (PicoQuant GmbH, Berlin, Germany). The fluorescent probe used was 2-dimethylamino-6-lauroilnaphthalene (laurdan) (Sigma-Aldrich, St. Louis, MO, USA). The binding of the probe with the membrane fraction was conducted by adding laurdan (diluted in methanol down to the final concentration of 10 μM) to the suspension of the fraction of mitochondria and chloroplasts. The suspension was incubated at 20 ± 2 °C for 10 min and analyzed with the use of the confocal microscope. Registration of images of our mitochondrial fractions (300 × 300 pixels) was conducted via the two channels (the wavelengths being 440 and 490 nm, respectively (I_exitation_ = 340 nm)).

### 4.5. Statistical Analysis

The experiments were conducted in the form of three independent replicates. Statistically significant differences between samples were revealed with the Mann–Whitney U test technique (*p* < 0.05) and represent M ± SD of three biological replicates. Letters above the bars indicate statistical meaning (*p* < 0.05). The data analysis was fulfilled with the use of SPSS17.0 (SPSS, Inc., Chicago, IL, USA), Statistica 6.0 (StatSoft, Inc., Tulsa, OK, USA) and Microsoft Excel 2007.

## 5. Conclusions

On the basis of the results obtained it may be stated that there is a reliable ground for the following conclusions: (i) DRMs of chloroplasts are enriched in cerebrosides (CERs), and sterols (STs) dominate in the mass of mitochondrial DRMs; (ii) the impact of salinity provokes obvious growth of the content of CERs in DRMs of chloroplasts; (iii) The FA composition, as well as the content of STs in DRMs of mitochondrion, was less affected by salinity.

It is possible to state that chloroplasts, mitochondria and perhaps individual sub-compartments (detergent-resistant membranes) of chloroplasts and mitochondria are involved in the processes of cell protection against (resistance to) salinity.

## Data Availability

Data related to the present paper are available upon request from the interested parties.
